# Application of virtual simulation situational model in Russian spatial preposition teaching

**DOI:** 10.3389/fpsyg.2022.985887

**Published:** 2022-09-16

**Authors:** Yanrong Gao, R. T. Kassymova, Yong Luo

**Affiliations:** ^1^Faculty of Philology and World Languages, Al-Farabi Kazakh Nation University, Almaty, Kazakhstan; ^2^Euro-Language's College, Zhejiang Yuexiu University, Shaoxing, China; ^3^Network and Educational Technology Center, Zhejiang Yuexiu University, Shaoxing, China

**Keywords:** virtual simulation, situation simulation, Russian teaching, spatial preposition, SSVEP-BCI

## Abstract

The purpose is to improve the teaching quality of Russian spatial prepositions in colleges. This work takes teaching Russian spatial prepositions as an example to study the key technologies in 3D Virtual Simulation (VS) teaching. 3D VS situational teaching is a high-end visual teaching technology. VS situation construction focuses on Human-Computer Interaction (HCI) to explore and present a realistic language teaching scene. Here, the Steady State Visual Evoked Potential (SSVEP) is used to control Brain-Computer Interface (BCI). An SSVEP-BCI system is constructed through the Hybrid Frequency-Phase Modulation (HFPM). The acquisition system can obtain the current SSVEP from the user's brain to know which module the user is watching to complete instructions encoded by the module. Experiments show that the recognition accuracy of the proposed SSVEP-BCI system based on HFPM increases with data length. When the data length is 0.6-s, the Information Transfer Rate (ITR) reaches the highest: 242.21 ± 46.88 bits/min. Therefore, a high-speed BCI character input system based on SSVEP is designed using HFPM. The main contribution of this work is to build a SSVEP-BCI system based on joint frequency phase modulation. It is better than the currently-known brain computer interface character input system, and is of great value to optimize the performance of the virtual simulation situation system for Russian spatial preposition teaching.

## Introduction

The earliest Russian Education in China began during the rule of Emperor Kangxi of the Qing Dynasty (1636–1912) and has a history of more than 300 years. Russian education and teaching were once brilliant throughout the history of China. Due to the changes in international relations, the economic recession in Russia, and the sharp decline in China-Russia trade, Russian students in Chinese colleges have decreased significantly. Russian education is also declining. There are many reasons for the fallback of school Russian education, mainly because of students' low interest in Russian learning (Khashimova et al., 2021; Panova et al., 2021; Vitalyevna, 2021). In China, English teaching has been popularized in primary schools. Thus, a sudden change from English to Russian learning in junior high school generates negative feelings among students. Secondly, some students are forced to learn Russian because of their parents' and teachers' decisions. Since it is against their own will, developing good Russian learning habits is difficult. Thirdly, some teachers lack a standard Russian pronunciation and adopt the traditional cramming mode, which is also difficult to mobilize students' learning enthusiasm. Therefore, teachers are responsible for cultivating students' interest in learning Russian (Li et al., 2021; Markova and Kvapil, 2021; Shaby et al., 2021).

Three-Dimensional (3D) virtual scene technology is an advanced visualization technology. It enables students to learn in an environment similar to the actual environment (Xiao-Dong and Hong-Hui, 2020; Zhao et al., 2020). All real-time data of “physical space” are collected through sensors, and display and more analysis, simulation, drilling, training and monitoring functions are realized in the “virtual presentation” environment with three dimensions. In this way , the production simulation in the virtual environment can be seamlessly integrated with the production in the reality. The virtual situation-based scene creation can open up a new Virtual Reality (VR) learning environment for language teaching. Also, it breaks through the application of Information Technology (IT) in teaching. The 3D virtual situation practical teaching system has changed the disadvantages of the traditional teaching model. It has brought students to the Russian world situation to feel the stimulation of foreign languages more intuitively. In addition, the 3D virtual situational practical teaching 6A (activity) system can solve the problem that students' language learning cannot be applied to a certain extent. The combination and cooperation of 3D technology and foreign language teaching is a brand-new teaching model, which has the internal power to change the learning environment and broad application space (Huang et al., 2018; Li et al., 2020; Luo, 2022). In the Virtual Simulation (VS) teaching situation, the combination of Brain-Computer Interface (BCI) and Artificial Intelligence (AI) can dynamically adjust educational tasks according to the personalized characteristics of subjects and their brain activities. This allows the education system to balance productivity with fatigue and boredom (Auccahuasi, 2021; Balderas et al., 2021; Wang, 2022). At the same time, the additional combination with VR is expected to provide users with an appropriate environment and expand the user experience (Papanastasiou et al., 2020).

Therefore, there is no case of using 3D virtual situation technology in Russian teaching in Russian or Chinese colleges. Let alone relevant research on the innovative Russian education environment. With regard to the problem of situation-based VS in Russian teaching, this work introduces the theory of situation perception and proposes a situation-driven Augmented Reality BCI (AR-BCI) interactive fusion system. BCI converts the electrophysiological signals of the central nervous system into messages and instructions and has an impact on the outside world. Thus, it realizes users' wishes similar to conventional neuromuscular channels. The innovation is that by designing an interactive interface module with machine context perception and an interactive means interface driven by human brain context cognition, the advantages of machine autonomous intelligence and human brain cognitive decision-making are fully exploited. Further, this work uses the Steady-State Visual Evoked Potential (SSVEP) to control the BCI and constructs an SSVEP-BCI system through the Hybrid Frequency Phase Modulation (HFPM) method. It is expected to construct a high-speed BCI character input system to promote the practice of BCI and its application in virtual situational teaching.

### Related work

Context is crucial for language teaching, but traditional language classroom teaching cannot provide a language context. Thus, it has certain limitations. Situation simulation teaching theory advocates that teaching activities should be carried out by constructing real situations. With the mutual penetration of VS technology and experimental teaching, virtual classroom, virtual simulation training base, and virtual training have been realized. They help promote systematic and standard development and enrich the equipment of VS experimental teaching. Also, they popularize immersive, experiential, and situational learning. Angelini and Muñiz (2021) set up different scenes of language training and used virtual technology to restore real situations, such as speeches and interviews. Their findings offered learners scenes of communication and interaction. Learners got familiar with the environment and adapted to interference, thus overcoming anxiety and psychological fear. The proposed method gave language communication real meaning and trained, cultivated, and improved students' oral ability and level. Kamhi-Stein et al. (2020) investigated using the hybrid reality simulation platform Mursion in language teaching projects. The results showed that the simulation reality method could reflect the teaching progress of the real classroom and enhance students' learning interests.

In constructing VS scenarios, through the implantable BCI, high-throughput neural signals can be directly obtained from the brain tissue. The information interaction channel between the central nervous system and the external physical world can be established. At present, many new and interesting applications have been produced. Implantable BCI is a key enabling technology to realize the integration of brain and machine. It is expected to realize the deep integration of biological intelligence and machine intelligence by establishing the direct interaction between brain and machine (Gao et al., 2021). Cattan et al. (2020) integrated the BCI based on P300 into the VR environment. The research achieved a high transmission rate and improved the user's game experience in the virtual environment. Shin et al. (2022) argued that information science should collect and feedback information in the brain and the system. Material science needed a new carrier for information transmission. Psychological science also needed to make bold assumptions and explore the expression of nerve, direct sense, and subconsciousness; Finally, a closed-loop BCI was formed by fusing multiple disciplines.

The above studies imply that integrating VS technology and foreign language experimental teaching is the general trend and is bound to make great achievements in future teaching practice. In the process of constructing the VS situation, it is necessary to further explore the Human-Computer Interaction (HCI) to present a more realistic language teaching scene. This work will focus on the specific aspects of Russian teaching and discuss the specific application of VS scenes in detail.

## Materials and methods

### Semantic correspondence of chinese and russian spatial prepositions and comparison of phrase syntax

The main purpose of Russian undergraduate teaching is to enable Russian Majors to use Russian as an auxiliary means to obtain the professional materials and provide necessary professional knowledge reserves for the future specialized applications of scientific and technical Russian and professional Russian, including language knowledge and language application ability. Russian course is an important basis for professional Russian courses to cultivate professional and technical Russian talents (Keihani et al., 2018). Optimizing the teaching quality of College Russian courses will guarantee high-quality professional Russian courses.

In Russian, the grammatical relationship between words and the grammatical function of words in sentences are mainly expressed through morphological changes. Russian is an Indo-European language with the most ancient morphological changes. Most nouns have 12 forms, and the singular and plural have six cases, respectively. Adjectives have more than 20 or even more than 30 forms. Moreover, singular masculine, neutral, feminine, and plural have six cases each, with short tails and comparative degrees. There can be one or two hundred verb forms, including aspect, tense, state, form, adjective, and adverb. Notional words can generally be divided into stem and suffix. The stem indicates the lexical meaning of a word. The ending of a word indicates grammatical meaning. Usually, an ending contains several grammatical meanings. There are many similarities between Russian and Chinese prepositions in grammatical meaning and syntactic function. Grammatically, Russian prepositions are equivalent to prepositions in Chinese but are expressed by different terms in the two languages (Cattan et al., 2020; Shin et al., 2022). Russian prepositions far outnumber Chinese prepositions. While analyzing the corresponding relationship between Chinese spatial prepositions and Russian spatial prepositions, researchers find that each Russian spatial preposition has rich ideographic meanings. For example, “B + the six case noun” can mean “in...”, and “Ha + the six case noun” can mean “on...”. By comparison, the Chinese have unique characteristics. In Chinese sentences, a preposition structure of “preposition + noun + Locatives” is considered a complete meaning. A single Chinese Preposition “在” cannot express a complete meaning. Russian prepositions are much more than Chinese prepositions and have much richer meanings. Thus, a Chinese preposition can be translated into multiple Russian prepositions (Baykalova et al., 2018; Galkina and Alexandra, 2019; Unlu, 2019). This one-to-many relationship is a difficult point for Chinese speakers to learn Russian. Learners can overcome the selection obstacles by finding similarities and differences using comparison. According to research, the prepositions such as “"在,” “向,” and “从” are used more frequently in Chinese spatial prepositions. In addition, prepositions such as “沿着” and “迎着” can find their grammatical equivalents in Russian, with multiple choices. Russian prepositions, such as “B, Ha, and y,” which express the spatial meaning of “in,” “on,” and “besides” in Chinese, can all be uniformly categorized into the Chinese preposition “在” structure. The corresponding relationship between the meaning of preposition “在” structure in Chinese and Russian prepositions is shown in Table 1.

**Table 1:** A comparison of the corresponding relationship between the Chinese preposition “在” structure to Russian propositions.

**”在“ structure in Chinese sentences**	**Russian preposition + required case**
在……里 (in something)	B+6, внyтри+2
在……上 (on something)	ha, под+5
在……下 (under something)	пд+5
在……附近 (near something)	около+2
在……中间 (in the middle of something)	среди+2
在……前面 (in front of something)	перед+5
在……某人那里 (in somebody's place)	y+2
在……后面 (in the back of something)	a+5
在……两者之间 (between A and B)	между+5
在……内部 (within something)	внyтри+2

Similarly, Russian prepositions like “K, BHyTpи, Πoд” that express the spatial meaning of “向/往/朝……去” (for/to/toward something), “向/往/朝……里” (into something), and “向/往/朝……下” (downward) in Chinese are uniformly classified into the Chinese preposition “向” structure. Table 2 shows the corresponding relationship between the preposition “向” structure in Chinese and multiple Russian prepositions.

**Table 2:** A comparison of the corresponding relationship between the Chinese preposition “向” structure and Russian prepositions.

**Chinese “向” preposition structure**	**Russian preposition + required case**
向……里 (into)	B+4
向……去、向……上面 (toward/upward)	Ha+4
向……下 (downward)	под+5
在……那边 (over)	sa+4

Russian words must change to a specific case before being combined with a prepositional to express a specific grammatical meaning. In Chinese “noun Locatives” prepositional phrases, nouns do not need to change cases. The same Russian spatial prepositions followed by different cases can express different grammatical meanings. For example, “B” + the fourth case indicates a direction, and “B” + the sixth case means the place. In contrast, the grammatical meaning of Chinese spatial prepositions is relatively stable. There are subtle differences between Chinese and Russian in using prepositions with similar meanings. Using similar but different examples to set up situations, foreign language learners can master spatial prepositions faster.

### Russian audio-visual oral teaching under virtual simulation

The 3D virtual situational teaching system can record and live broadcast the synthetic video in real-time for other teachers and students to observe and comment. The system will release the recorded synthetic courses through the teaching resource management system (Hsu et al., 2021). Students can copy them to their computers for repeated speculation and learning to improve their practical training ability. The potential of 3D virtual situation training teaching applications needs to be tapped and effectively opened (Chahine and Uetova, 2021; Zoda, 2022). Language learning needs continuous practice in a specific context. Learning in a rich language environment can significantly improve students' learning efficiency. 3D virtual situational training and teaching can effectively help students solve problems, improve the learning environment, and enrich teaching forms (Almousa et al., 2019; Ahir et al., 2020; Philippe et al., 2020).

**Figure 1:**
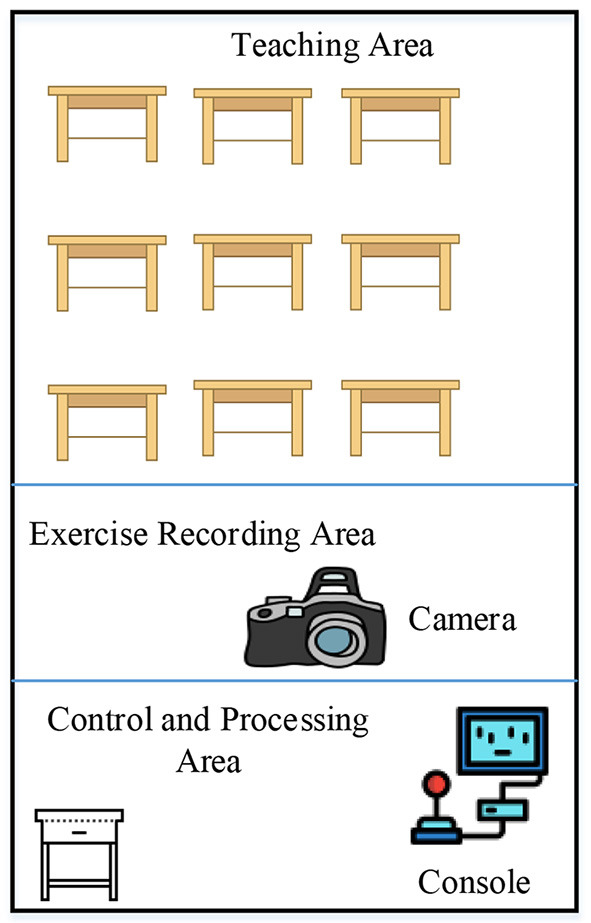
Structure of 3D virtual recording and broadcasting room for Russian teaching.

As a symbol of thinking and communication, language is an organic combination of pronunciation, grammar, and semantics produced in a given context. Virtual Reality (VR) technology can provide the necessary environment for language learning; its connection with Russian teaching is mainly reflected in vocabulary and grammar teaching. People can improve vocabulary accumulation by memorizing Russian words through visual senses. The course teaching must be done in the 3D virtual recording and broadcasting room. The structure of the 3D virtual recording and the broadcasting room is shown in Figure 1. Teachers use simulation technology to create a vivid virtual language learning scene and form a complete 3D virtual activity. The synthesized video will eventually be delivered to students' mobile phones and tablet terminals. Teachers use simulation technology to create a vivid virtual language learning scene to form a complete 3D virtual activity. The synthesized video will eventually be delivered to students' mobile phones and tablet terminals. 3D technology can help recognize the problem of “stating direction, orientation, and path” in Russian in a virtual scene. Learners can understand the teaching difficulty of Russian motion verbs with different prefixes through the function of hearing and vision.

### Virtual situation AR-BCI system

**Figure 2:**
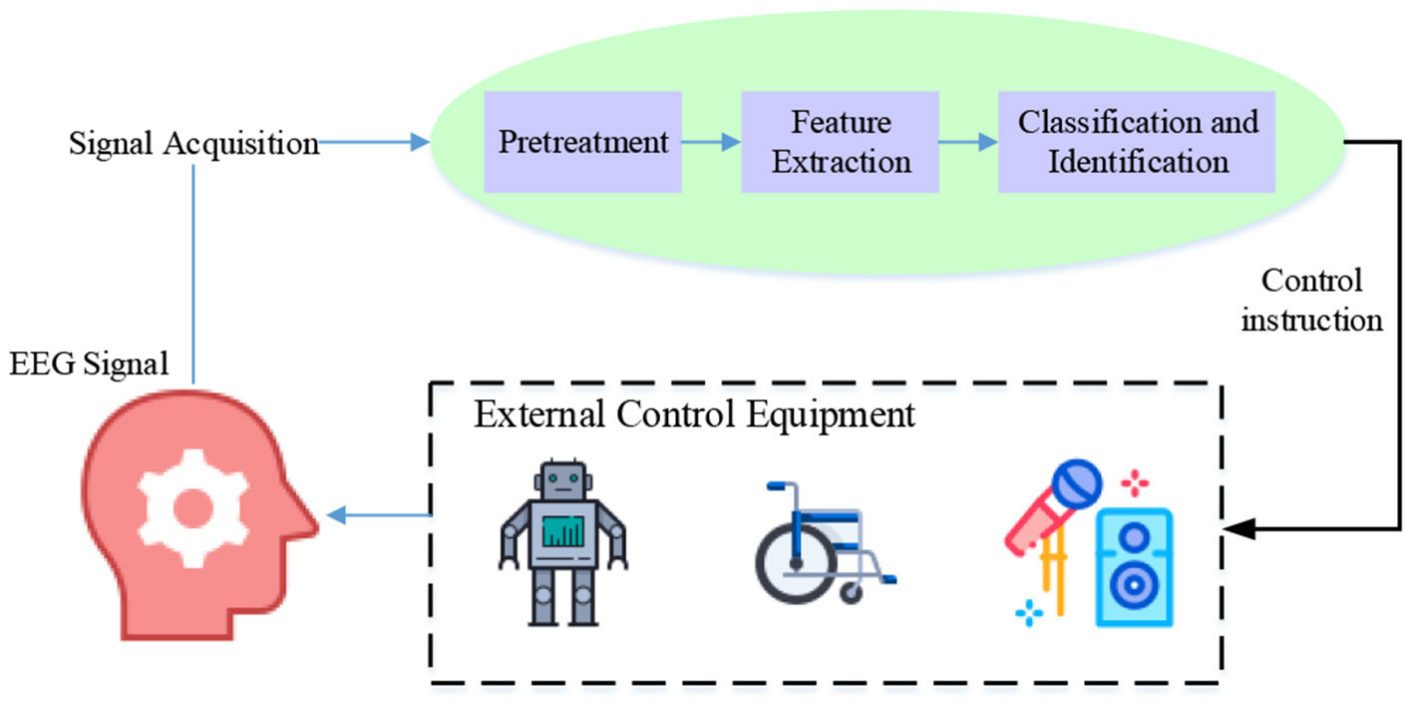
BCI technical workflow.

A complete BCI process includes four steps: signal acquisition, information decoding and processing, signal output/execution, and feedback (Wang P. et al., 2018). BCI can collect or feedback signals through electricity, magnetism, light, and sound. Electroencephalogram (EEG) technology is a mainstream exploration direction. There are many ways to collect central nerve signals to monitor brain activity, including EEG, functional Near-infrared Spectroscopy (fNIRS), and functional Magnetic Resonance Imaging (fMRI). Feedback techniques also include electricity, magnetism, sound, and light. BCI is a brain signal detection technology. It decodes a specific brain thinking activity and converts it into a command signal that computers and other devices can understand. This signal is then output to drive wearable devices on tissues and organs to act according to brain ideas. The key link is to correctly analyze the sensory behavior signals of the brain (Jensen and Konradsen, 2018; Ke et al., 2020; Arpaia et al., 2021). BCI technical workflow is shown in Figure 2. A variety of brain signals (such as EEG, magnetoencephalogram, functional magnetic resonance, functional near infrared spectroscopy, cortical EEG and local field potential, etc.) designed in the signal acquisition process can be used as signals of brain computer interface. The next signal processing is the core work of brain computer interface, which decodes human intention by analyzing and processing signals. The signal processing of BCI includes preprocessing, feature extraction, and pattern classification. After feature extraction, a classifier should be established to classify the features.

**Figure 3:**
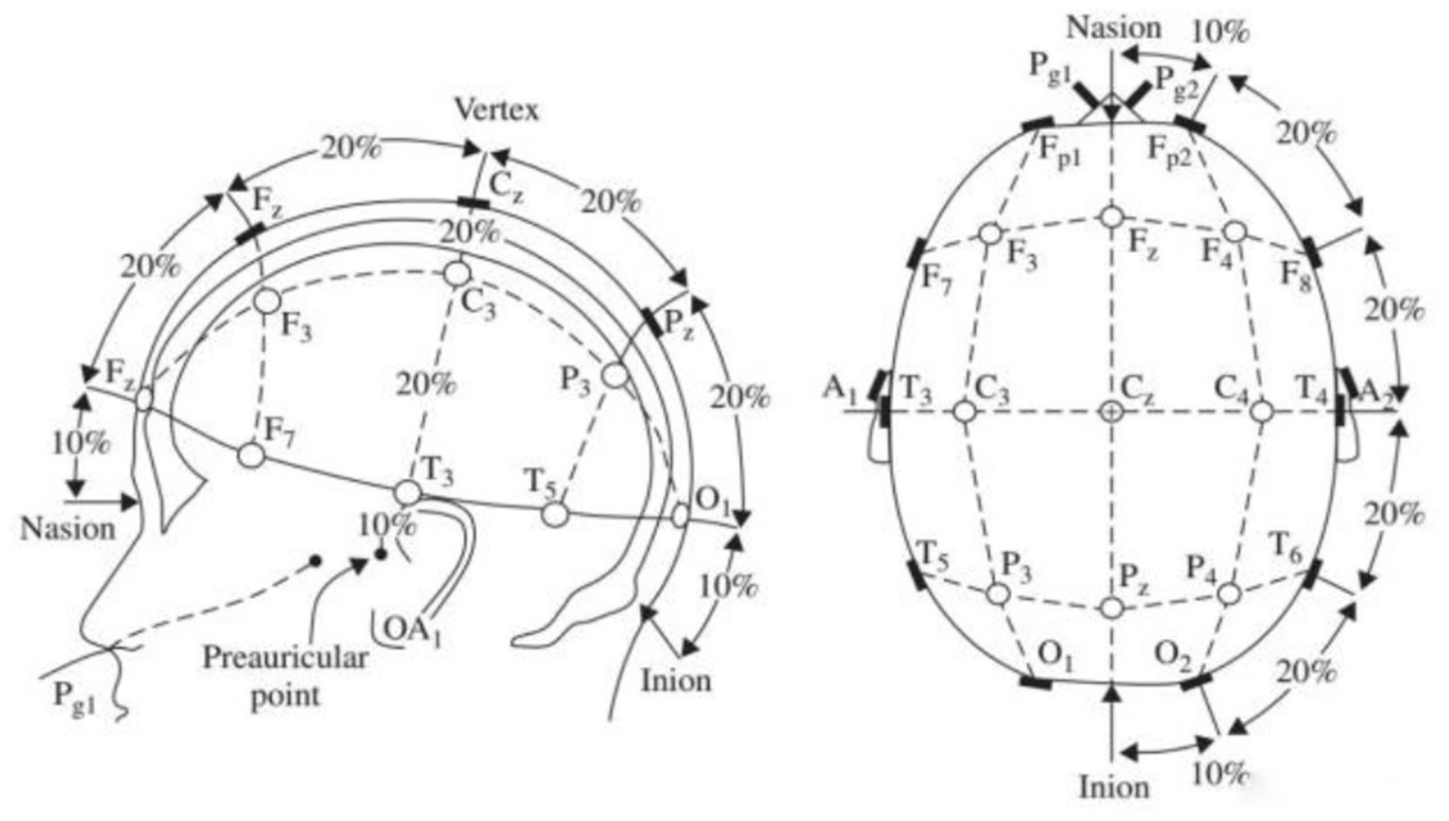
International 10–20 standard electrode lead diagram.

EEG acquisition is the critical step of BCI. The effect of acquisition, signal strength, stability, and bandwidth directly determine the subsequent processing and output. Changes in the membrane potential of central neurons in the brain will produce spikes or action potentials. The ion movement transmitted between synapses of nerve cells will form field potentials. These neurophysiological signals can be collected and amplified by external connection or implantation of microelectrodes in the cerebral cortex motor nerves. The subjects wore electrode caps and used conductive paste to increase the conductivity between the cerebral cortex and electrodes. The international 10–20 standard electrode leads are shown in Figure 3. Take 8 electrodes on the left and right sides, respectively, the midpoint of the forehead (Fz), central point (CZ), vertex (Pz), and two ear electrodes on the anterior and posterior positions, a total of 21 electrodes. Brain activities are transformed into electrical signals through signal processing. It removes interference waves and other signals, classifies and processes targets, and converts them into corresponding signals that can be output. Signal output transmits the collected and processed EEG signals to the connected equipment or feeds them back to the terminal machine as instructions (Kim et al., 2021; Zhang et al., 2022). The equipment generates actions or displays contents once the signal is executed. The participants will feel that the brain waves generated in the first step have been executed through vision, touch, or hearing and trigger the feedback signal.

SSVEP is an EEG signal based on frequency domain features. Therefore, this work uses Canonical Correlation Analysis (CCA) to extract the EEG signal features. When a constant frequency external visual stimulation is applied, the neural network consistent with the stimulation frequency or harmonic frequency will produce resonance. It will lead to significant changes in brain potential activity at the stimulation frequency or harmonic frequency, resulting in SSVEP signals. SSVEP signal is manifested in EEG signal, and the spectral peak can appear on the stimulation frequency or harmonic in the power spectrum. By analyzing the frequency corresponding to the peak of the detection spectrum, the stimulus source of the visual gaze of the subject can be detected to identify the intention of the subject.

Suppose the number of frequency stimuli is *M*; *X* is the collected multi-channel EEG signal; *Y* is the sine and cosine reference signal corresponding to the frequency of visual stimuli. Its structure can be expressed as:


(1) $$Y_{m}=\left[\begin{array}{l} \sin\left(2\pi f_{m}t\right)\\ \cos\left(2\pi f_{m}t\right)\\ ...\\ \sin\left(2\pi Hf_{m}t\right)\\ \cos\left(2\pi Hf_{m}t\right) \end{array}\right],t=\frac{1}{F},\frac{2}{F}...\frac{P}{F}$$

In Equation (1) *f*_*m*_, *H*, *F*, and *P* refer to the stimulation frequency, the number of harmonics, the sampling rate, and the number of signal samples.

Vectors *Wx* and *Wy* can maximize the correlation between vectors *x* and *y*. The typical correlation coefficient ρ of *X* and can be expressed as:


(2) $$\begin{array}{r} \rho =\underset{Wx,Wy}{\max}\frac{E\left[xy^{T}\right]}{\sqrt{E\left[xx^{T}\right]E\left[yy^{T}\right]}} \end{array}$$

Further, the visual stimulation frequency $\hat{f}$ of SSVEP can be expressed as:


(3) $$\begin{array}{r} \hat{f}=\underset{f_{m}}{\arg\max}\rho_{m} \end{array}$$

The basic principle of HoloLens is the near-eye 3D diffraction information display technology. It projects the obtained virtual content from the front micro projector to the photoconductive lens and then into the human eye (Chen et al., 2020; Apicella et al., 2022). HoloLens can carry out *XYZ*-three-axis modeling of the surrounding space and recognize the user's gestures through multiple cameras and sensors. The modeling operation of this 3D coordinate axis also makes it possible for multiple HoloLLens to share virtual objects and interact with each other. That is, user B can see the virtual scenery generated by user A.

**Figure 4:**
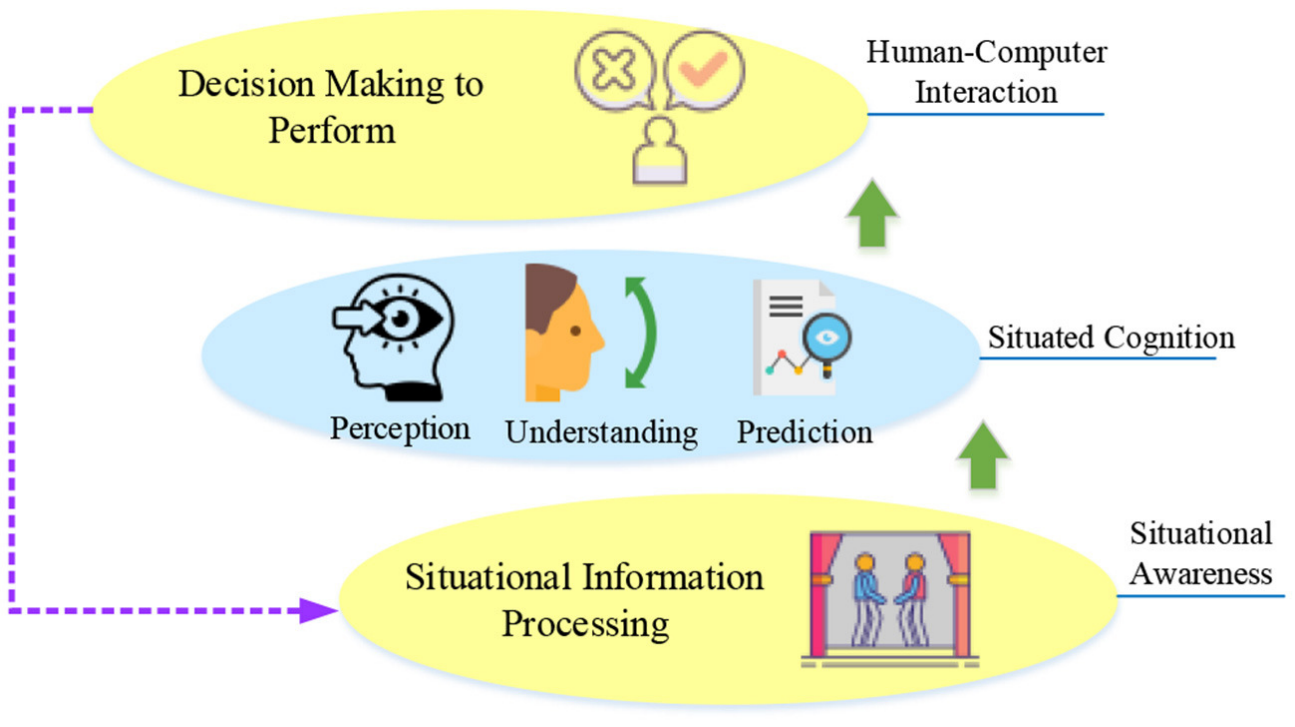
Situation-based HCI system.

Situational awareness refers to the operator's perception and understanding of the current system environment and predicting future system situational changes. Endsley constructed a three-level theoretical model of situational awareness: perception, understanding, and prediction. The situation-based Human-Computer Interaction (HCCI) system is shown in Figure 4. The system includes situational information collection and processing, and application services. The purpose is to present the data processing results to users as information and to produce user cognition to make decisions or behavioral responses. Situational cognition emphasizes that users generate decisions and judgments about tasks or systems in this context based on situational awareness. Users complete the HCI and form the interactive feedback by outputting behaviors and executing actions through the machine.

### Frequency phase hybrid coding method in BCI

Control application is a major branch of BCI. It concerns reasonably designing and matching BCI control tasks. It ensures the efficiency of BCI and good HCI experience, the reliability and safety of the control system, and the coordination and unity of BCI and control equipment. Brain nerve cells will produce potential activity changes on different stimuli. These changes are most obvious in or near the primary visual cortex, namely, the occipital lobe. This change in EEG signals is called the Visual Evoked Potential (VEP) (Al Janabi et al., 2020; Moro et al., 2021). VEP is divided into transient VEP and Steady-State VEP (SSVEP) according to the characteristics of stimulus frequency. Due to individual differences, both the BCI system based on motor imagination and the P300-BCI system need to train the subjects for a long time. Thus, it greatly reduces the ease of use of the BCI system. By comparison, the SSVEP-BCI system needs less or no training and has strong adaptability.

When a constant-frequency external visual stimulus is applied, the Neural Network (NN) consistent with the stimulus frequency or harmonic frequency will produce resonance. This results in significant changes in brain potential activity at the stimulus frequency or harmonic frequency, thus generating SSVEP signals.

Physiologically, each brain part has its division of labor. The sensory, motor, and cognitive modules of different cortical regions are independent, as shown in Figure 4. However, functional modules cooperate with each other to form an organic whole. When the brain processes perceptual information, many modules work in parallel. BCI system based on SSVEP signal judges brain thinking activity by detecting EEG signal of occipital visual area. The acquisition system in SSVEP-BCI can obtain the current SSVEP from the user's brain and then know which module the user is currently watching. A crucial part of the SSVEP-BCI system is the stimulation module that induces SSVEP. The brain will generate SSVEP with different frequencies. Nowadays, there are three kinds of stimulators used to realize stimulation modules, namely Liquid Crystal Display (LCD), Cathode Ray Tube (CRT), and Light Emitting Diode (LED) (Wang M. et al., 2018; Chai et al., 2020; Thielen et al., 2021).

The SSVEP-BCI system multi-frequency permutation coding includes the following steps. It places all available frequencies of the stimulator in the coding frequency set. Each stimulation module is periodically coded in a unique frequency arrangement. A coding cycle consists of two or more time segments. This work aims to construct a high-speed BCI character input system using a hybrid frequency-phase coding method. The idea of filter bank analysis is introduced into canonical correlation analysis, a filter bank canonical correlation analysis is proposed, and a 40-target brain computer interface character input system based on frequency coding is designed. A BCI character input system based on SSVEP is designed using hybrid frequency-phase coding. Frequency coding usually adopts an equal interval frequency coding target:


(4) $$x_{n}\left(t\right)=\sin\left\{2\pi \left[f_{0}+\left(n-1\right)\Delta f\right]t\right\}$$

In Equation (4), *f*_0_ is the smallest frequency used. ▵f denotes the frequency interval. means the index of the target.

Introducing equally spaced phases into frequency coding can increase the difference in frequency coding targets:


(5) $$x_{n}\left(t\right)=\sin\left\{2\pi \left[f_{0}+\left(n-1\right)cf\right]t+\phi_{0}+\left(n-1\right)\Delta \phi \right\}$$

In Equation (5), ϕ_0_ and ▵ϕ respectively represent the initial phase and phase interval of the target at the minimum frequency.

The zero phase segment data are further circularly shifted. The SSVEP with different phase interval values can be:


(6) $$\begin{array}{c} \overset{⌢}{X}(f_{k},{\bar{\phi }}_{k},n)=\bar{X}(f_{k},0,n+\frac{(2\pi -\phi_{k})\times f_{k}}{2\pi \times f_{k}}) \end{array}$$

In the online simulation, the BCI system performance is evaluated using the Leave-One-Out Cross-Validation (LOO-CV). The Cross-Validation (CV) method in target recognition generates training reference signals from training data. Classification accuracy and Information Transfer Rate (ITR) indicate the BCI system performance. The amount of information output for each judgment is:


(7) $$\begin{array}{r} B=\log_{2}^{N}+p\log_{2}^{p}+\left(1-p\right)\times \log_{2}\left(\frac{1-p}{N-1}\right) \end{array}$$

In Equation (7), refers to the number of targets. *p* is the recognition accuracy. The ITR can be expressed as:


(8) $$\begin{array}{r} ITR=B\left(60/T\right) \end{array}$$

In Equation (8), *T* indicates the time required for each instruction output.

The experimental data are collected in the AR-SSVEP paradigm, which includes four groups. A total of 120 trials are collected from four stimulus targets in each group. The sampling rate of EEG data is 1 KHz, and the band-pass filter is 0.5–100 Hz. Four stimulus layouts AR-Pos1~AR-Pos4 are set in AR-SSVEP. The horizontal interval of the same stimulus target in the adjacent layouts is 128.

## Results

### Performance results of VS situation AR-SSVEP

**Figure 5:**
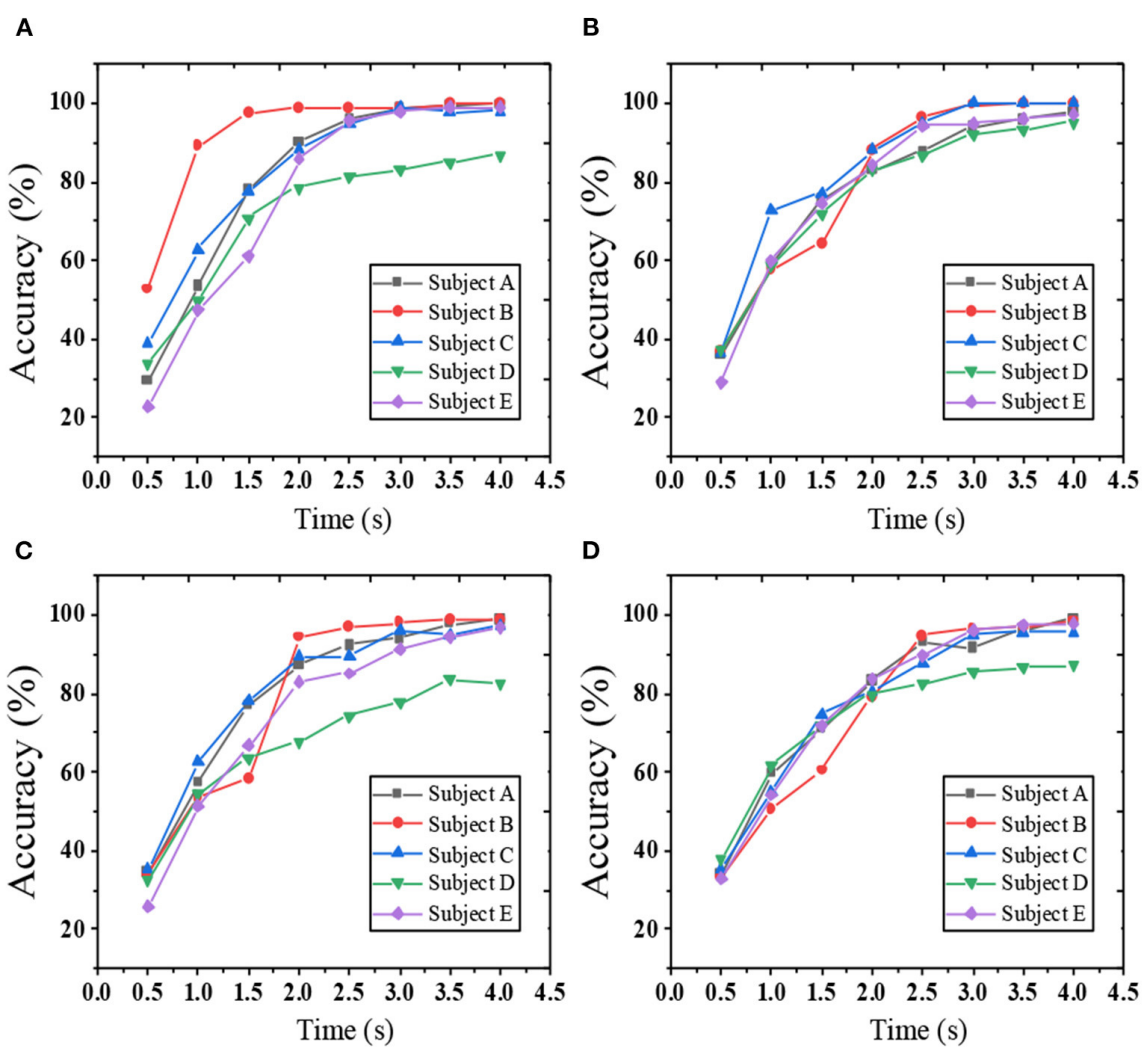
Correlation coefficients of a single-trial SSVEP of a subject [**(A)** position 1; **(B)** position 2; **(C)** position 3; **(D)** position 4].

The optimal stimulus location layout is obtained through the recognition accuracy of HoloLens at different locations. According to the experimental results, the recognition accuracy and ITR of five subjects' offline and simulated online SSVEP are analyzed. Under the four layouts of AR-SSVEP, the stimulation duration is 0.5–4 s, and the step length is 0.5 s. The classification accuracy is calculated for four positions at different times; the results are shown in Figure 5. Apparently, with the increase in data length, the classification accuracy of each subject gradually increases. The classification accuracy of the four layouts is different. Suppose the standard threshold of classification accuracy is 90%. When the time window length is 1, 2, and 3s, the number of subjects who reach the threshold in the AR-Pos2 layout is higher than in the other three positions.

### Test results of mixed frequency-phase recognition method

**Figure 6:**
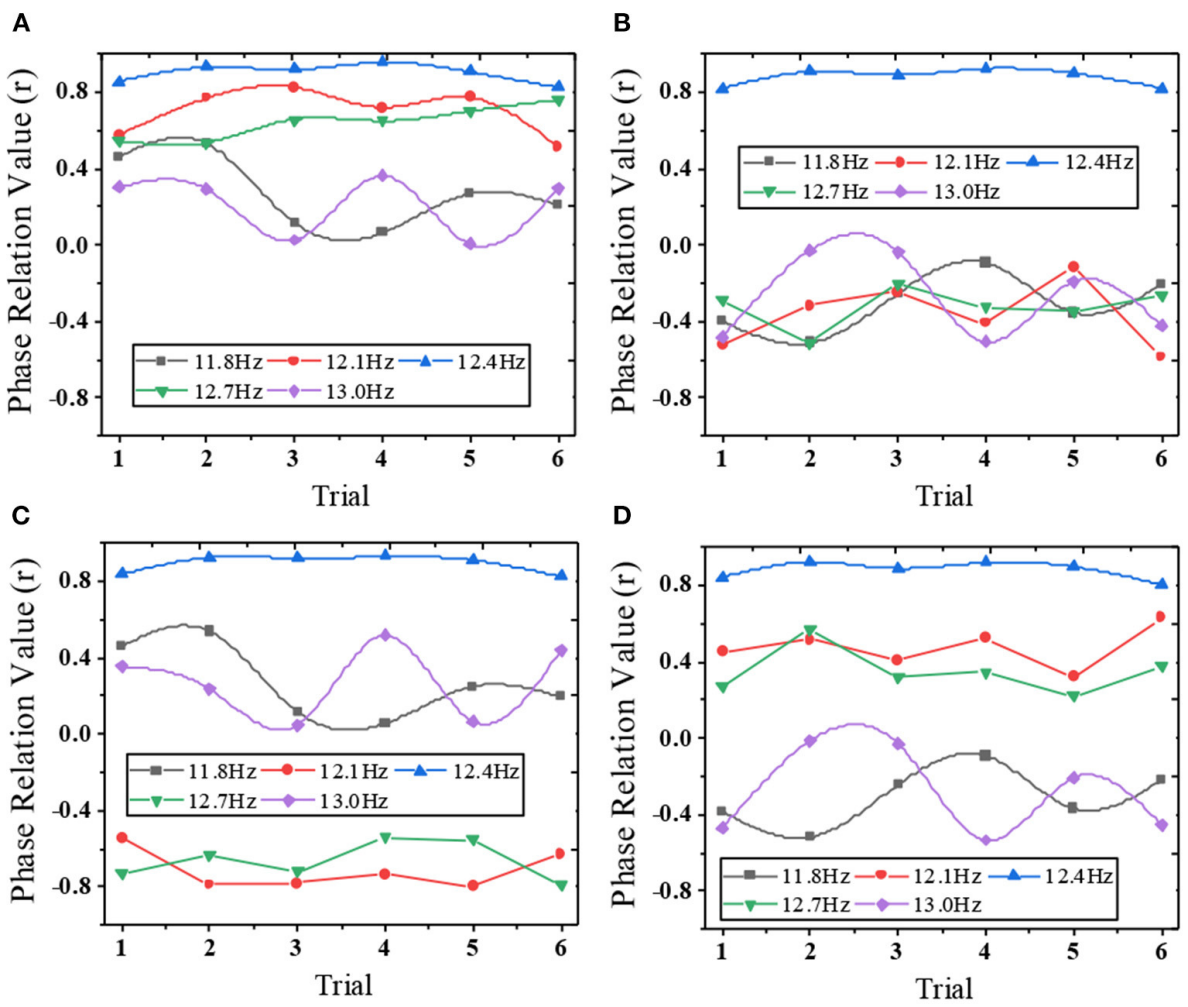
Correlation coefficients between SSVEP and adjacent frequency for a single test at 12.4 Hz [**(A)** Δϕ = 0; **(B)** Δϕ = 0.5π; **(C)** Δϕ = π; **(D)** Δϕ = 1.5π).

Figure 6 shows the correlation coefficients of a single-trial SSVEP of a subject. The numerical results are highly consistent with the theoretical pattern from the stimulus signal. Four different phase interval values lead to different phase patterns. Under different phase interval values, the correlation coefficients between 12.4 Hz and adjacent frequencies are significantly different. When the phase interval is , the maximum correlation coefficient is obtained at the target frequency (12.4 Hz, 0.7). This shows that the identification accuracy of SSVEP can be significantly improved by introducing Phase Modulation (PM) into the HFPM.

**Figure 7:**
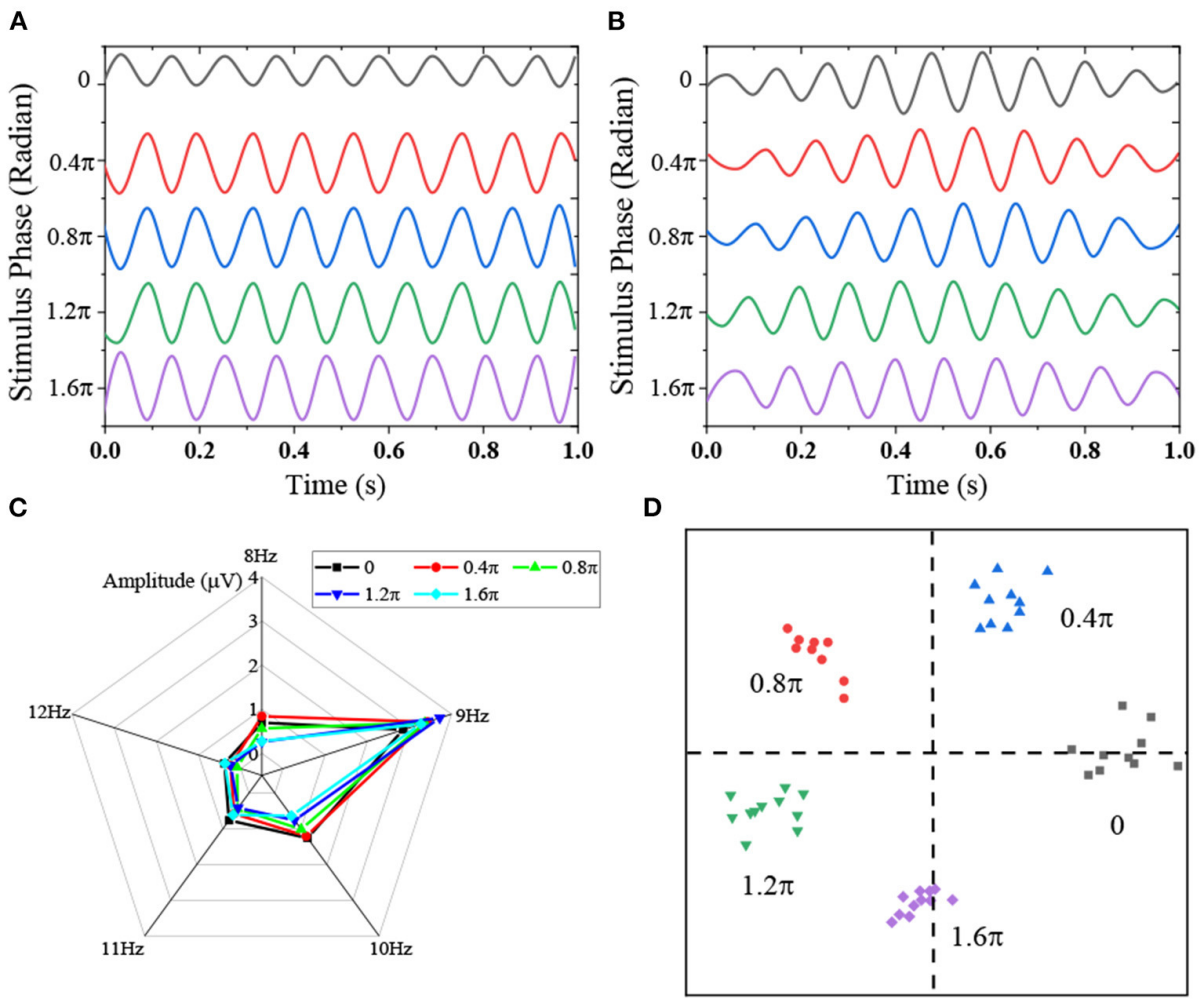
Mixed frequency-phase coding [**(A)** induced SSVEP under different stimulation phases; **(B)** time-domain waveform under different stimulation phases; **(C)** amplitude spectrum of induced SSVEP; **(D)** complex spectral scatter of induced SSVEP].

The presentation of stimulus signals plays a vital role in SSVEP-BCI. Next, 40 stimulus signals are generated by the sampling sine coding method. Figure 7 shows the time domain waveforms of stimulus signals corresponding to different phases at the same frequency (9 Hz) and the corresponding average SSVEP of a single subject. Obviously, the amplitude peaks of SSVEP induced by different phases at the same frequency are all at 9 Hz. Thus, the sampling sine coding method can induce robust SSVEP signals and accurately encode frequency information.

**Figure 8:**
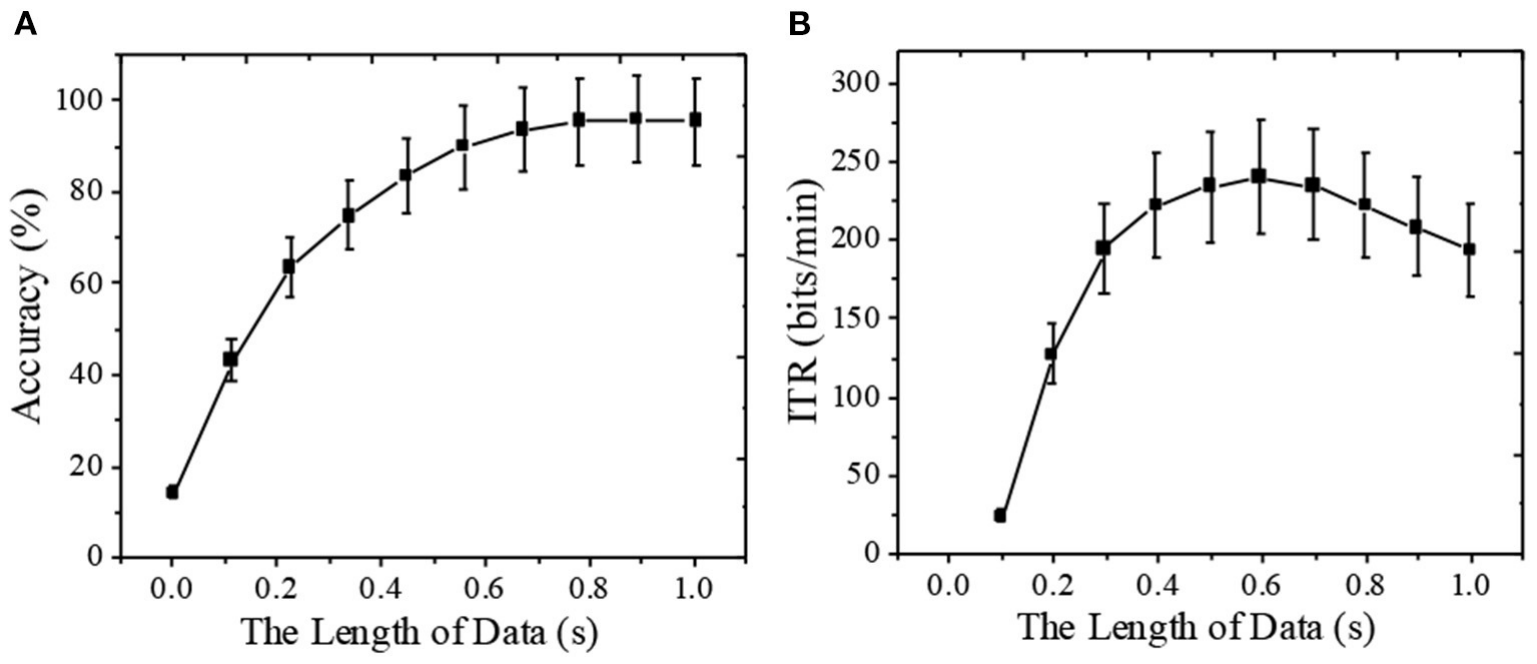
Performance of online BCI system under hybrid frequency-phase coding with different data lengths [**(A)** classification accuracy; **(B)** ITR].

Subsequently, the performance of the online BCI system with different data lengths is further studied. The system's classification accuracy and ITR under HFPM (40 class hours) are calculated. Figure 8 shows the performance of the simulated online BCI system under different data lengths in the mixed coding paradigm. The results in Figure 8 suggest that the classification accuracy of the system will increase with the increase of data length. The accuracy and ITR of joint coding paradigm also increase with the increase of data length. Moreover, the classification accuracy is still significantly higher than the opportunity level when the data length is short. The ITR reaches the highest when the data length is 0.6-s, reaching 242.21 ± 46.88 bits/min. In the actual use, the target recognition time of the online BCI system should be optimized by comprehensively considering the accuracy and ITR.

## Conclusion

Under the teaching picture of Unipus 3.0, teaching resources, teaching scenes, the teaching practice, and the interaction model between the core teaching elements have transformed the foreign language digital education from the local optimization to a comprehensive upgrade in the digital intelligence age. The promotion of VS experiment teaching 2.0 should strengthen the basic construction from three aspects: the accumulation of basic teaching materials, the exploration of experimental teaching rules, and the construction and development of a simulation system. At the same time, it should learn from intelligent VS to realize the innovative development of liberal arts experiment teaching and open up new ways in future teaching practice.

3D VS situational teaching is a high-end visual teaching technology in which the creation of virtual situations is the primary link. Considering that the language teaching of foreign languages needs a real context, and the 3D VS situation technology can realize this idea. This work chooses the Russian spatial preposition teaching as an example to study the key technologies of situation-based VS. In constructing VS scenes. A BCI machine can control the human brain. The machine can give feedback to the ideas of the human brain. Further, it carries out a systematic study of the AR-BCI system in virtual situations. The CCA method is used to extract the EEG features. Next, the SSVEP-BCI frequency recognition method is proposed, and the SSVEP-BCI system is designed and implemented. Finally, a novel hybrid frequency-phase coding method is proposed to improve the average ITR. The results show that the recognition accuracy of SSVEP can be significantly improved by introducing PM into the HFPM. This work still has some limitations. For example, it does not consider that different design characteristics in virtual situational teaching will bring different interface design effects, which will affect user cognition and operation performance. In the future, the design of interactive interface should combine visual perception and cognitive psychology, such as analyzing the display of graphics and text in the interactive interface, thus evaluating the impact of interface design performance on user performance.

## Contributions

In order to improve the teaching quality of Russian spatial prepositions, this work constructs an SSVEP-BCI system based on HFPM and explores the 3D VS situational teaching system. The proposed system is superior to the currently known BCI character input system. It has important value for optimizing the performance of the VS situation system to improve the overall experience of Russian teaching.

## Data availability statement

The original contributions presented in the study are included in the article/supplementary material, further inquiries can be directed to the corresponding author/s.

## Ethics statement

Ethical approval for this study and written informed consent from the participants of the study were not required in accordance with local legislation and national guidelines.

## Author contributions

All authors listed have made a substantial, direct, and intellectual contribution to the work and approved it for publication.

## Funding

This work was supported by Provincial first-class Curriculum Construction project Basic Russian 2 for undergraduate colleges and universities of Zhejiang Provincial Department of Education (Zhejiang Education Office Letter [2020] No. 77).

## Conflict of interest

The authors declare that the research was conducted in the absence of any commercial or financial relationships that could be construed as a potential conflict of interest.

## Publisher's note

All claims expressed in this article are solely those of the authors and do not necessarily represent those of their affiliated organizations, or those of the publisher, the editors and the reviewers. Any product that may be evaluated in this article, or claim that may be made by its manufacturer, is not guaranteed or endorsed by the publisher.
